# Ethnobotanical study on herbal tea drinks in Guangxi, China

**DOI:** 10.1186/s13002-023-00579-3

**Published:** 2023-03-31

**Authors:** Tingyu Long, Renchuan Hu, Zhuo Cheng, Chuangui Xu, Qimin Hu, Qingling Liu, Ronghui Gu, Yunfeng Huang, Chunlin Long

**Affiliations:** 1 Key Laboratory of Plant Resource Conservation and Germplasm Innovation in Mountainous Region (Guizhou University), Ministry of Education, Guiyang, 550025 China; 2 School of Liquor and Food Engineering, Guizhou University, Guiyang, 550025 China; 3 Guangxi Key Laboratory of Traditional Chinese Medicine Quality Standards, Guangxi Institute of Traditional Medicine and Pharmaceutical Science, Nanning, 530022 China; 4 College of Life and Environmental Sciences, Minzu University of China, Beijing, 100081 China; 5 Key Laboratory of Ecology and Environment in Minority Areas (Minzu University of China), National Commission of Ethnic Affairs, Beijing, 100081 China; 6 Key Laboratory of Ethnomedicine (Minzu University of China), Ministry of Education, Beijing, 100081 China; 7 College of Life Sciences, Guizhou University, Guiyang, 550025 China

**Keywords:** Ethnobotany, Herbal tea, Medicinal effects, Traditional knowledge, Guangxi

## Abstract

**Background:**

Herbal tea drinks, different from classical *Camellia* beverages, are a wide variety of herbal drinks consumed for therapeutic purposes or health promotion. Herbal tea is widely consumed in Guangxi. However, the documentation on the plants for herbal tea and their related health benefits is still limited.

**Methods:**

An ethnobotanical survey was conducted in 52 villages and 21 traditional markets in Guangxi from 2016 to 2021. Semi-structured interviews, key informant interviews, and structured questionnaires were applied to obtain ethnobotanical information of herbal tea, in which 463 informants had participated. Relative frequency of citation (RFC) and cultural food significance index (CFSI) were used to evaluate the most culturally significant herbal tea plants, and informant consensus factor (ICF) was applied to assess the agreement among informants.

**Results:**

This study recorded 155 herbal tea species belonging to 49 families. The most commonly used parts included leaf (27.61%), whole plant (22.09%), branch and leaf (19.02%), and flower (13.50%). The most frequent preparation method of herbal tea was decoction. Herbal tea was very popular in Guangxi, attributing to its therapeutic value, special odor, and good taste. There are 41 health benefits classified into eight categories. Among them, clearing heat was the most medicinal effects. Local people had high consistency in tonic, removing cold and cough, improving blood circulation, and clearing heat away. Based on CFSI values of each species, the most culturally significant herbal tea species were *Siraitia grosvenorii* (Swingle) C. Jeffrey ex A. M. Lu & Zhi Y. Zhang, *Plantago asiatica* L., *Gynostemma pentaphyllum* (Thunb.) Makino, *Zingiber officinale* Roscoe, *Pholidota chinensis* Lindl., and *Morus alba* L.

**Conclusion:**

Herbal tea is a valuable heritage that carries the local people’s traditional knowledge, like health care and religious belief. The recorded herbal tea species in this study possess tremendous potential for local economic development in the future. Further research on efficacy evaluation and product development of herbal tea species is necessary.

**Supplementary Information:**

The online version contains supplementary material available at 10.1186/s13002-023-00579-3.

## Background

Tea (*Camellia sinensis* (L.) Kuntze) is among the world’s most widely consumed beverages and embodies numerous economic, health, and cultural values [1–3]. Over two-thirds of the world’s population drank tea, and approximately two billion cups of tea are consumed daily [4]. In general, the plant species used to make various tea, including Green Tea, White Tea, Black Tea, and Pu’er Tea, belong to the subgeneric group Thea of the genus *Camellia* [5, 6]. However, many other plant species, which are not belong to *Camellia*, have been widely used as herbal tea or substitute tea [7–10].

Herbal tea, defined as water-based infusions/decoctions prepared with herbal ingredients other than *Camellia sinensis*, is used medicinally by indigenous and local peoples for improved nutrition, prevention, and treatment of health problems [11–13]. Usually, herbal tea may consist of one or several plant species prepared using poach, infusion, or maceration [14]. They are typically made from different plant parts, such as leaves, stems, fruits, flowers, seeds, and barks, intended to achieve a specific purpose, including relaxation, rejuvenation, or relief from a specific condition [15]. Nowadays, herbal tea is becoming increasingly popular worldwide due to their diverse biological properties (*e.g.,* fragrance, taste, antioxidant properties, and so on), cultural and religious principles, and complementary effects [16–18].

China has a long history, rich biodiversity, and diverse ethnic culture. Over the long history, different linguistic groups have accumulated traditional knowledge of using herbal tea to treat diseases [17]. It is estimated that a total of 782 plant species are used as herbal tea in China, and 82% of the total species are used in Southern China [11]. For example, 222 ethno-taxa corresponded to 238 botanical taxa (species, varieties, or subspecies) that were recorded as herbal tea in the Lingnan region of Southern China [17].

Guangxi, an autonomous region of multiethnic groups living together with Zhuang people as the main group, is in the southwest of China. Due to the unique geographical location and superior climatic condition, Guangxi has rich natural resources [18]. Especially for plant species, Guangxi has 8562 known species of wild vascular plants, ranking top three in the country after Yunnan and Sichuan. Herbal tea drinks are popular in Guangxi and play a crucial role in protecting their health during long-term life practices to defend the heat and humidity [19]. Our previous ethnobotanical investigation found that herbal tea in Guangxi is fully popular as a daily practice by local people [20–22]. However, there have been only sporadic reports on the research of herbal tea in Guangxi, and these studies have not investigated the herbal tea comprehensively, especially lack of evaluation methods using quantitative indices [7]. Guangxi herbal tea has a long history, and there are many kinds of herbal tea exhibiting their own characteristics in different regions of Guangxi. These characteristics and traditional knowledge of herbal tea are urgently needed to be protected due to habitat loss, influence from mainstream culture, and modernization [23, 24]. Therefore, ethnobotanical research is necessary to investigate and document the herbal tea in Guangxi to inform conservation efforts of biocultural diversity toward supporting environmental and human well-being. On the other hand, the study and development of those herbal tea may bring new health benefits to human society or make better economic value for local communities.

To record and better understand the traditional knowledge and characteristics of Guangxi herbal tea, we carried out a comprehensive ethnobotanical investigation across Guangxi and conducted systematic evaluation on the plant species, cultural significance, health consistency, regional characteristics, and the challenges of the herbal tea in Guangxi. Given this, the objectives of this study are as follows: (1) How many herbal species have been used traditionally; (2) How and why the local people used the herbal species; (3) How to evaluate the importance of herbal species to local people and which plants are special. Obviously, this study will facilitate the protection and development of Guangxi’s herbal tea.

## Methods

### Study area

Guangxi Zhuang Autonomous Region is located in the south of China, between 104°28′–112°04′ E and 20°54′–26°23′ N, including 14 prefecture-level cities and 111 county-level administrative regions [20]. It covers an area of 237,600 km^2^. It is located at low latitude, with the tropic of cancer crossing the central part, the tropical ocean to the south, the Nanling Mountains to the north, and the Yun-Gui Plateau to the west. It is a tropical and subtropical monsoon climate zone. The complex and varied geographical environment and the excellent climate provide suitable conditions for rich biodiversity. Meanwhile, Guangxi is an autonomous region inhabited by many ethnic groups, including Zhuang (31.36%), Yao (3.7%), Miao (1.1%), Dong (0.7%), Mulam (0.4%), and Maonan (0.17%) [25]. They have created an effulgent art and culture, especially the tea culture [19].

In addition, the previous ethnobotanical studies on herbal tea in Chaoshan [26], Fujian [27], and Taiwan [28], were selected for comparison with Guangxi in order to illustrate whether geographical and cultural differences affected the choice and use of herbal tea species in Guangxi. Chaoshan region lies in eastern Guangdong (a province next to Guangxi) and has a subtropical marine climate. Because of its abundant rainfall and sunshine, herbal tea drinks are very popular in this region for clearing heat [26]. Two Han branches (Chaoshanese speaking Chaoshan dialects and Hakka speaking Hakka dialects) are the main populations living in the Chaoshan region [29]. Fujian, which is adjacent to Chaoshan region, is located in southeast China. The sultry and humid subtropical monsoon climate in Fujian contributes its rich biodiversity, including many herbal tea species [27]. The Han Chinese including Hakka people are the main population in Fujian. Taiwan faces Fujian across the sea and has tropical and subtropical monsoon climate, which lead to hot and humid weather in summer, and local people consume herbal tea to clear heat and remove dampness [28]. In Taiwan, the population is composed of Han people (97%, including Hakka), aboriginals (2%), and others (1%) [28, 30].

### Ethnobotanical survey and data collection

**Fig. 1:**
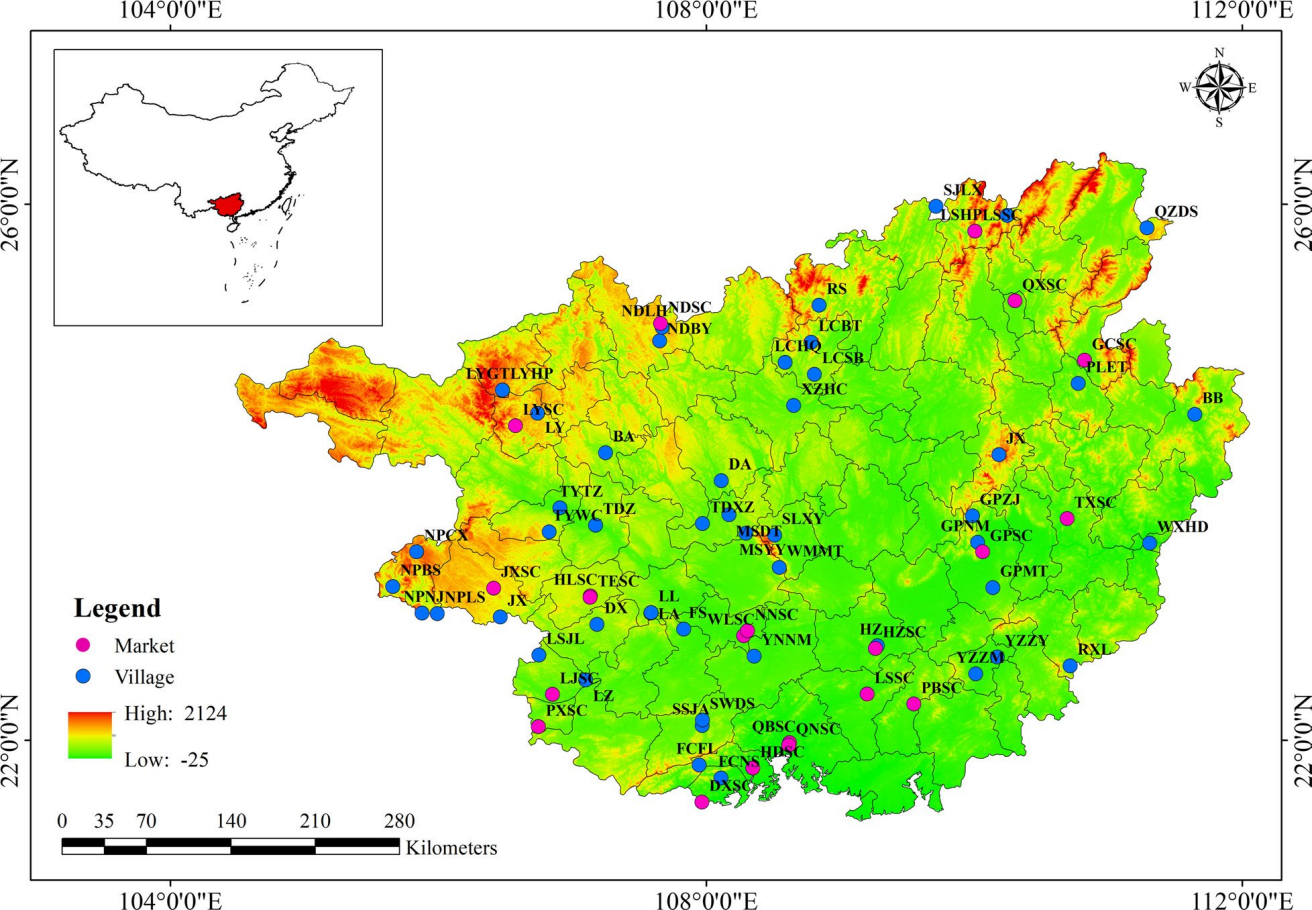
Locations of the ethnobotanic investigation on herbal tea in Guangxi

**Fig. 2:**
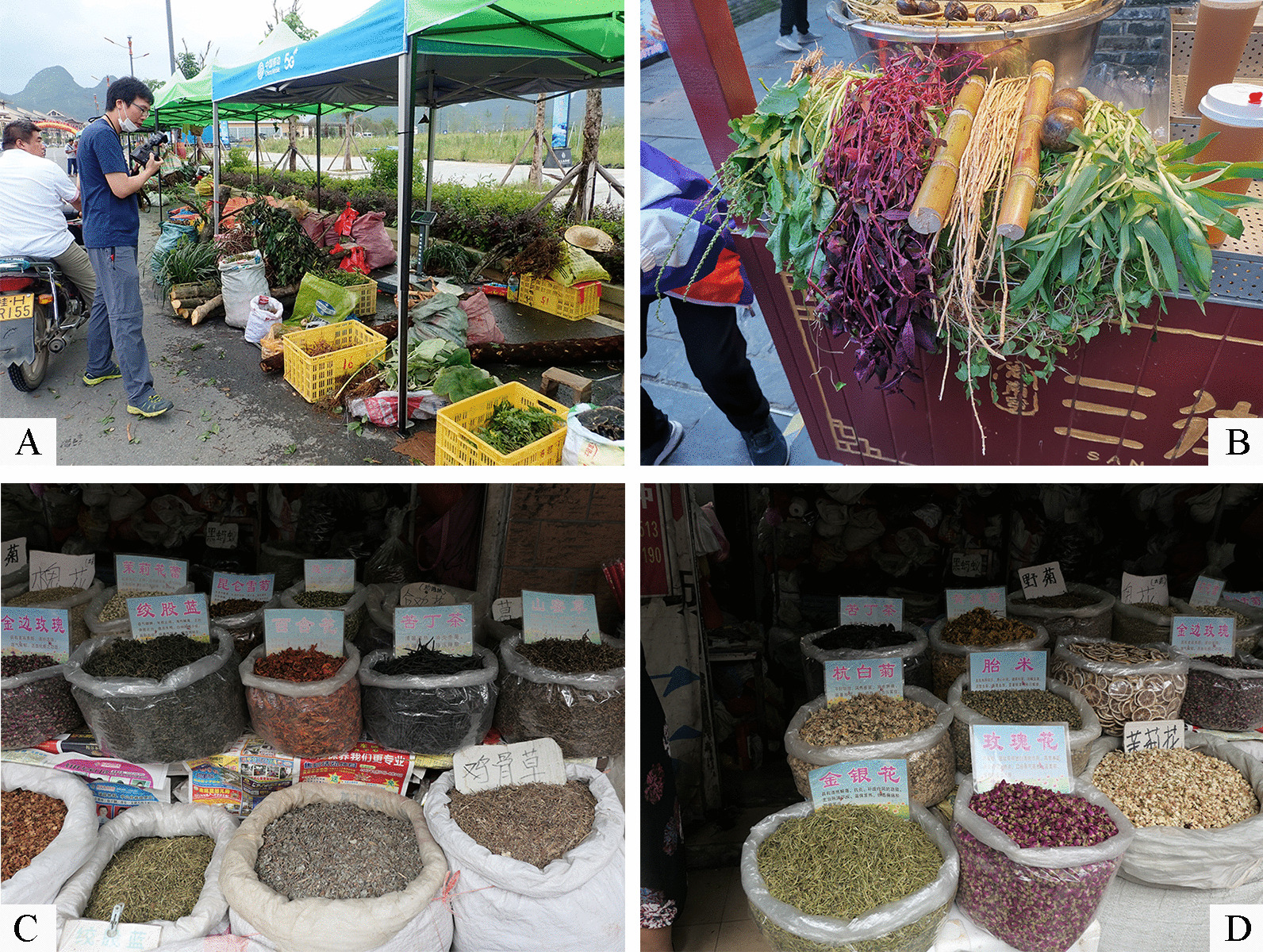
**A** Ethnobotanical investigation of herbal tea; **B–D** herbal tea plants in medicinal markets

Field surveys were conducted based on the five surveys between October 2016 and May 2021. The Snowball sampling method was mainly used for the participant selection, and the semi-structured interview was mainly used to collect related information about herbal tea. Before each interview, prior informed consent was requested throughout the study [31]. After obtaining permission, various participants (farmers 23%, vendors 25%, village leaders 12%, religious leaders 4%, and traditional healers 36%) were interviewed. Based on the records from references, suggestions from local government, our knowledge and experience, and the results from snowball interviews, 51 villages and 21 traditional markets in Guangxi were selected as study locations (Fig. 1, Additional file 1: Table S1). A total of 463 informants were interviewed between 21 and 70 years old from these study locations to record plants used for herbal tea and document traditional knowledge of their habitats, used parts, medicinal effects, and preparation methods (Fig. 2), in which the habitats, including cultivated, wild, and cultivated or wild, were defined according to whether or not the plants grown with artificial care. Of the informants, 80% were over 45 years old, most had a low education level, and these informants were almost equally male and female. Product samples and voucher specimens were collected from markets, mountains, forests, and farming fields. In addition, photographs to record all plant species and gathering activities were taken simultaneously. Voucher specimens of all plants available during field investigations were collected and deposited in the herbarium of Guangxi Institute of Traditional (GXMI), Guangxi Academy of Traditional Medical and Pharmaceutical Sciences. Product samples, voucher specimens, and photographs were identified and confirmed referring to *Flora of China*, *Flora of Guangxi,* and botanical Web sites (e.g., http://www.tropicos.org/, http://www.cvh.ac.cn/search, http://www.plant.csdb.cn/).The botanical names were listed following *Plants of the World Online* database (https://powo.science.kew.org). Finally, the identified specimens were confirmed by other taxonomists from GXMI and completed the inventory of plant species consumed as herbal tea.

### Data analysis

Data analysis was carried out to evaluate how important and indispensable the herbal tea species are to local healthcare and daily diets. The taxonomic diversity, used parts, preparation methods, and categories of health-promoting were counted and analyzed. Moreover, three indices were applied to furtherly estimate the importance of certain species to the local community, which were named the relative frequency of citation (RFC), the informant consensus factor (ICF), and the cultural food significance index (CFSI).

The RFC was performed to quantify the use frequency of certain species, which was determined using the following formula:

Relative frequency of citation: ${\text{RFC }} = {\text{ FC }}/{\text{ N}}$.

FC refers to the number of respondents who mentioned a particular herbal tea plant, and N represents the number of informants participating in the survey [32, 33].

The ICF was used to measure the agreement among informants on the health-promoting effects of each herbal tea plant. The value was calculated following the formula:

Informant Consensus Factor: ${\text{ICF}} = \, \left( {{\text{Nur }} - {\text{ Nt}}} \right) \, / \, \left( {{\text{Nur }} - \, 1} \right)$.

Nur is the number of informants reporting a certain health-promoting effect, and Nt is the total number of herbal tea plants used for the particular health-promoting effect [34].

The CFSI elaborated to evaluate the cultural significance of herbal tea plants by following the formula:

Cultural food significance index:${\text{CFSI }} = {\text{ QI }} \times {\text{ AI }} \times {\text{ FUI }} \times {\text{ PUI }} \times {\text{ MFFI }} \times {\text{ TSAI }} \times {\text{ FMRI }} \times 10^{ - 2}$.

Seven indexes in the formula expressed the frequency of quotation (mention) by informants (QI), the availability of a plant (AI), the frequency of utilization (FUI), the used parts of the plant (PUI), multi-functional food use (MFFI), the taste score appreciation index (TSAI), and the food-medicinal role score (FMRI), respectively [35–37].

## Results

### Diversity of herbal tea plants in Guangxi

Our investigations showed that 155 plant species were used to make herbal tea in Guangxi. Ethnobotanical information of each species, including family, scientific name, Chinese name, habit, parts used, preparation and uses, habitat, materials status (dry or fresh), health-promoting effects, RFC, CFSI, and voucher number, is listed in Table 1.

**Table 1:** Local herbal tea plants in Guangxi Province

No.	Family	Scientific name	Chinese name	Habit	Part used	Preparation and uses	Habitat	Materials status	Health-promoting effects	RFC	CFSI	Voucher number
1	Annonaceae	*Alphonsea hainanensis* Merr. & Chun	Hai nan teng chun 海南藤春	Tree	Branch and leaf	Decoction	Wild	Dry or Fresh	–	0.032	9.9	HYF160114002
2	Apiaceae	*Centella asiatica* (L.) Urb	Ji xue cao 积雪草	Herb	Whole plant	Decoction	Wild	Dry or Fresh	Clearing heat away and detoxifying	0.294	1958.4	HYF1501106010
3	Apocynaceae	*Melodinus fusiformis* Champ. ex Benth	Cha teng 茶藤	Liana	Branch and leaf	Decoction	Wild	Dry	–	0.006	6.8	HYF180108033
4	Apocynaceae	*Plumeria rubra* L	Ji dan hua 鸡蛋花	Shrub	Flower	Soak	Cultivated	Dry or Fresh	Clearing heat away, moistening lungs and relieving cough	0.13	121.5	HYF190705016
5	Aquifoliaceae	*Ilex asprella* (Hook. & Arn.) Champ. ex Benth	Mei ye dong qing 梅叶冬青	Shrub	Root and stem	Decoction	Wild	Dry	Clearing heat away and detoxifying	0.108	66	HYF180201008
6	Aquifoliaceae	*Ilex hainanensis* Merr	Hai nan dong qing 海南冬青	Tree	Leaf	Decoction	Wild	Dry	Relieving summer heat, lower cholesterol	0.125	76.6	HYF180201010
7	Aquifoliaceae	*Ilex jingxiensis* Y. F. Huang & M. X. Lai	Jing xi dong qing 靖西冬青	Tree	Leaf	Decoction	Wild	Dry	Clearing heat away and detoxifying	0.022	5.9	HYF170921001
8	Aquifoliaceae	*Ilex kudingcha* C. J. Tseng	Ku ding cha 苦丁茶	Tree	Leaf	Soak	Cultivated and wild	Dry	Clearing heat away and detoxifying	0.637	1380.6	HYF170921003
9	Aquifoliaceae	*Ilex pentagona* S. K. Chen, Y. X. Feng & C. F. Liang	Wu leng ku ding cha 五棱苦丁茶	Tree	Leaf	Soak	Wild	Dry	Clearing heat away and detoxifying	0.06	32.8	HYF170921005
10	Aquifoliaceae	*Ilex pubescens* Hook. & Arn	Mao dong qing 毛冬青	Shrub	Leaf	Decoction	Wild	Dry	Clearing heat away and detoxifying	0.091	83.2	HYF160126011
11	Aquifoliaceae	*Ilex rotunda* Thunb	Tie dong qing 铁冬青	Tree	Bark	Decoction	Wild	Dry	Clearing heat away and detoxifying	0.359	219.1	HYF160126012
12	Araliaceae	*Heptapleurum heptaphyllum* (L.) Y. F. Deng	E zhang chai 鹅掌柴	Tree	Root	Decoction	Wild	Dry	Clearing heat away and detoxifying	0.244	223.7	HYF171101003
13	Araliaceae	*Heptapleurum minutistellatum* (Merr. ex H. L. Li) Y. F. Deng	Xing mao ya jiao mu 星毛鸭脚木	Tree	Root	Decoction	Wild	Dry	Clearing heat away and detoxifying	0.14	128.7	HYF171101004
14	Araliaceae	*Panax notoginseng* (Burkill) F. H. Chen ex C. Chow & W. G. Huang	San qi 三七	Herb	Flower	Soak	Cultivated	Dry	Clearing heat away, detoxification, lowering the blood pressure	0.112	46.8	HYF221023002
15	Asteraceae	*Artemisia anomala* S. Moore	Qi hao 奇蒿	Herb	Branch and leaf	Decoction	Wild	Dry	Clearing heat away, diuresis, improving blood circulation	0.05	1408.3	HYF171028005
16	Asteraceae	*Chrysanthemum indicum* L	Ye ju 野菊	Herb	Flower	Soak	Wild	Dry	Clearing heat away and detoxifying, improving eyesight, lowering the blood pressure	0.704	874.8	HYF181014011
17	Asteraceae	*Launaea acaulis* (Roxb.) Babc. ex Kerr	Guang jing shuan guo ju 光茎栓果菊	Herb	Whole plant	Decoction	Wild	Dry or Fresh	Clearing heat away and detoxifying, moistening lungs and relieving cough	0.095	34.3	HYF160413016
18	Asteraceae	*Taraxacum mongolicum* Hand.-Mazz	Pu gong ying 蒲公英	Herb	Whole plant	Decoction	Cultivated or wild	Dry or Fresh	Clearing heat away and detoxifying, diuresis	0.175	20.2	HYF171031002
19	Begoniaceae	*Begonia fimbristipula* Hance	Zi bei tian kui 紫背天葵	Herb	Whole plant	Decoction	Wild	Dry	Detoxification, relieving cough, improving blood circulation, improving immunity	0.071	96.5	HYF171028007
20	Calycanthaceae	*Chimonanthus nitens* Oliv	Shan la mei 山蜡梅	Shrub	Leaf	Decoction	Wild	Fresh	Clearing heat away and detoxifying	0.017	5.2	HYF181014010
21	Caprifoliaceae	*Lonicera confusa* DC	Hua nan ren dong 华南忍冬	Liana	Flower	Soak	Cultivated or wild	Dry or Fresh	Clearing heat away and detoxifying	0.011	338.6	HYF171117003
22	Caprifoliaceae	*Lonicera hypoglauca* Miq	Gu xian ren dong 菰腺忍冬	Liana	Flower	Soak	Cultivated or wild	Dry or Fresh	Clearing heat away and detoxifying	0.492	18.7	HYF171117007
23	Caprifoliaceae	*Lonicera macrantha* (D. Don) Spreng	Da hua ren dong 大花忍冬	Liana	Flower	Soak	Wild	Dry or Fresh	Clearing heat away and detoxifying	0.104	156.4	HYF171117005
24	Caprifoliaceae	*Lonicera macranthoides* Hand.-Mazz	Hui zhan mao ren dong 灰毡毛忍冬	Liana	Flower	Soak	Cultivated or wild	Dry or Fresh	Clearing heat away and detoxifying	0.341	7.3	HYF171117010
25	Chloranthaceae	*Sarcandra glabra* (Thunb.) Nakai	Cao shan hu 草珊瑚	Shrub	Branch and leaf	Decoction	Wild	Dry	Clearing heat away and detoxifying	0.38	1689.6	HYF151121016
26	Cucurbitaceae	*Gynostemma compressum* X. X. Chen & D. R. Liang	Bian guo jiao gu lan 扁果绞股蓝	Liana	Whole plant	Decoction	Wild	Dry	Clearing heat away and detoxifying	0.032	35.1	HYF180112014
27	Cucurbitaceae	*Gynostemma guangxiense* X. X. Chen et D. H. Qin	Guang xi jiao gu lan 广西绞股蓝	Liana	Whole plant	Decoction	Wild	Dry	Clearing heat away and detoxifying	0.019	21.1	HYF170319007
28	Cucurbitaceae	*Gynostemma longipes* C. Y. Wu	Chang geng jiao gu lan 长梗绞股蓝	Liana	Whole plant	Decoction	Wild	Dry	Clearing heat away and detoxifying	0.032	35.1	HYF170319010
29	Cucurbitaceae	*Gynostemma pentaphyllum* (Thunb.) Makino	Jiao gu lan 绞股蓝	Liana	Whole plant	Soak	Wild	Dry	Clearing heat away and detoxifying, relieving cough, expectorant	0.562	3244.8	HYF170319011
30	Cucurbitaceae	*Gynostemma laxum* (Wall.) Cogn	Guang ye jiao gu lan 光叶绞股蓝	Liana	Whole plant	Decoction	Wild	Dry	Clearing heat away and detoxifying	0.006	7	HYF170319008
31	Cucurbitaceae	*Momordica charantia* L	Ku gua 苦瓜	Liana	Peel	Decoction	Cultivated	Dry	Relieve summer heat, improving eyesight, detoxification	0.292	364.5	HYF180108040
32	Cucurbitaceae	*Siraitia grosvenorii* (Swingle) C. Jeffrey ex A. M. Lu & Zhi Y. Zhang	Luo han guo 罗汉果	Liana	Fruit, flower	Soak	Cultivated	Dry	Clearing heat away and moistening lungs	0.773	5370	HYF151019008
33	Ebenaceae	*Diospyros kaki* L.f	Shi 柿	Tree	Leaf	Decoction	Cultivated	Dry	Aiding digestion	0.032	39.6	HYF190727001
34	Fabaceae	*Abrus precatorius* L	Xiang si zi 相思子	Liana	Leaf	Soak	Wild	Dry	Moistening lungs, clearing heat away, diuresis	0.363	725.8	HYF190706006
35	Fabaceae	*Callerya speciosa* (Champ. ex Benth.) J. Compton & Schrire	Mei li ya dou teng 美丽崖豆藤	Liana	Tuber	Decoction	Wild	Dry	Strong body	0.004	5.9	HYF180211011
36	Fabaceae	*Chamaecrista mimosoides* (L.) Greene	Han xiu cao jue ming 含羞草决明	Herb	Seed	Soak after stir-fry	Cultivated	Dry	Clearing heat away and detoxifying, diuresis, aid digestion	0.035	8.3	HYF181014008
37	Fabaceae	*Chamaecrista nictitans* (L.) Moench	Duan ye jue ming 短叶决明	Herb	Seed	Soak after stir-fry	Wild	Dry	Clearing liver heat and improving eyesight, aid digestion	0.026	3.1	HYF181014009
38	Fabaceae	*Pueraria montana* (Lour.) Merr	Ge 葛	Liana	Flower	Decoction	Wild	Dry	Refreshing, alleviate a hangover, alleviate a hangover	0.328	513	HYF190705023
39	Fabaceae	*Senna sophera* (L.) Roxb	Huai ye jue ming 槐叶决明	Herb	Seed	Soak after stir-fry	Wild	Dry	Clearing liver heat and improving eyesight, aid digestion	0.048	17.2	HYF151019006
40	Fabaceae	*Senna tora* (L.) Roxb	Jue ming 决明	Herb	Seed	Soak after stir-fry	Cultivated or wild	Dry	Clearing liver heat and improving eyesight, aid digestion	0.067	80.6	HYF151019007
41	Fabaceae	*Tadehagi pseudotriquetrum* (DC.) H. Ohashi	Man jing hu lu cha 蔓茎葫芦茶	Shrub	Branch and leaf	Decoction	Wild	Dry or Fresh	Relieving cough and reducing sputum	0.032	19.8	HYF171031005
42	Fabaceae	*Tadehagi triquetrum* (L.) H. Ohashi	Hu lu cha 葫芦茶	Shrub	Branch and leaf	Decoction	Wild	Dry or Fresh	Relieving cough and reducing sputum, diuresis, relieve summer heat	0.097	243	HYF171031001
43	Fagaceae	*Lithocarpus litseifolius* (Hance) Chun	Mu jiang ye ke 木姜叶柯	Tree	Leaf	Soak	Wild	Dry	Helping produce saliva and slake thirst, relieve summer heat	0.551	1377	HYF191112022
44	Helwingiaceae	*Helwingia chinensis* Batalin	Zhong hua qing jia ye 中华青荚叶	Shrub	Branch and leaf	Decoction	Wild	Dry	Clearing heat away	0.006	3.1	HYF180110012
45	Hypericaceae	*Cratoxylum cochinchinense* (Lour.) Blume	Huang niu mu 黄牛木	Tree	Branch and leaf	Soak	Wild	Dry	Heatstroke prevention, clearing away heat	0.127	368.2	HYF181120018
46	Hypericaceae	*Cratoxylum formosum* subsp. *pruniflorum* (Kurz) Gogelin	Hong ya mu 红芽木	Tree	Branch and leaf	Soak	Wild	Dry	Clearing heat away, relieve summer heat, anti-diarrhea	0.335	644.8	HYF181122003
47	Hypericaceae	*Hypericum japonicum* Thunb	Di er cao 地耳草	Herb	Whole plant	Decoction	Wild	Dry or Fresh	Clearing heat away, detoxification	0.125	229.7	HYF180110017
48	Juglandaceae	*Cyclocarya paliurus* (Batalin) Iljinsk	Qing qian liu 青钱柳	Tree	Branch and leaf	Decoction	Wild	Dry or Fresh	Clearing heat away, lowering the blood pressure	0.307	255.6	HYF181122012
49	Juglandaceae	*Engelhardia roxburghiana* Lindl	Huang qi 黄杞	Tree	Branch and leaf	Decoction	Wild	Dry	Clearing heat away and detoxifying, help produce saliva and slake thirst, relieve summer heat, decreasing blood glucose	0.365	912.6	HYF161104003
50	Lamiaceae	*Agastache rugosa* (Fisch. & C. A. Mey.) Kuntze	Huo xiang 藿香	Herb	Branch and leaf	Decoction	Cultivated or wild	Dry or Fresh	Relieving summer heat	0.123	256.5	HYF190813004
51	Lamiaceae	*Clerodendrum infortunatum* L	Bai hua deng long 白花灯笼	Herb	Root	Decoction	Wild	Dry	Clearing heat away, relieving cough, detoxification, and detumescence	0.026	15.9	HYF190312032
52	Lamiaceae	*Elsholtzia ciliata* (Thunb.) Hyl	Xiang ru 香薷	Herb	Branch and leaf	Decoction	Wild	Dry or Fresh	Diuresis, clearing heat away, relieve summer heat	0.048	105.6	HYF161104002
53	Lamiaceae	*Isodon lophanthoides* (Buch.-Ham. ex D. Don) H. Hara	Xian wen xiang cha cai 线纹香茶菜	Herb	Whole plant	Decoction	Wild	Dry or Fresh	Clearing heat away and detoxifying	0.052	64.8	HYF160413006
54	Lamiaceae	*Mentha canadensis* L	Bao he 薄荷	Herb	Branch and leaf	Soak	Cultivated or wild	Dry	Clearing heat away and detoxifying	0.039	194.4	HYF180108034
55	Lamiaceae	*Orthosiphon aristatus* (Blume) Miq	Shen cha 肾茶	Herb	Branch and leaf	Decoction	Cultivated	Fresh	Clearing heat away and diuresis	0.339	1017.4	HYF190312030
56	Lamiaceae	*Platostoma palustre* (Blume) A. J. Paton	Liang fen cao 凉粉草	Herb	Whole plant	Decoction	Cultivated or wild	Dry	Clearing heat away, relieve summer heat, diuresis	0.149	1117.8	HYF180108036
57	Lamiaceae	*Premna microphylla* Turcz	Dou fu chai 豆腐柴	Shrub	Leaf	Decoction	Wild	Dry or Fresh	Clearing heat away and detoxifying, detumescence	0.052	37.4	HYF190705019
58	Lamiaceae	*Prunella vulgaris* L	Xia ku cao 夏枯草	Herb	Whole plant	Decoction	Wild	Dry or Fresh	Clearing heat away and diuresis	0.069	249.6	HYF190705020
59	Lamiaceae	*Vitex negundo* L. var. *cannabifolia* (Sieb. & Zucc.) Hand.-Mazz	Mu jing 牡荆	Shrub	Branch and leaf	Decoction	Wild	Dry or Fresh	Clearing heat away, aid digestion	0.032	6.6	HYF180118012
60	Lamiaceae	*Vitex quinata* (Lour.) F. N. Williams	Shan mu jing 山牡荆	Tree	Branch and leaf, fruit	Decoction	Wild	Dry or Fresh	Clearing heat away, lowering the blood pressure	0.022	8.8	HYF151121013
61	Lauraceae	*Cinnamomum burmannii* (Nees & T. Nees) Blume	Yin xiang 阴香	Tree	Bark	Decoction	Wild	Dry	Expelling wind and removing cold	0.024	39.6	HYF171028025
62	Lauraceae	*Cinnamomum jensenianum* Hand.-Mazz	Ye huang gui 野黄桂	Tree	Branch and leaf	Decoction	Wild	Dry	Improving blood circulation, removing cold	0.013	10.8	HYF190312028
63	Lauraceae	*Neocinnamomum delavayi* (Lecomte) H. Liu	Xin zhang 新樟	Tree	Stem	Decoction	Wild	Dry	Anti-diarrhea, headache, protection against the cold	0.006	2.7	HYF171114011
64	Loranthaceae	*Helixanthera parasitica* Lour	Li ban ji sheng 离瓣寄生	Shrub	Whole plant	Decoction	Wild	Dry or Fresh	Determined by the host plants	0.048	158.4	HYF180110011
65	Loranthaceae	*Macrosolen cochinchinensis* (Lour.) Tiegh	Qiao hua 鞘花	Shrub	Whole plant	Decoction	Wild	Dry or Fresh	Determined by the host plants	0.086	324	HYF180111053
66	Loranthaceae	*Scurrula parasitica* L	Hong hua ji sheng 红花寄生	Shrub	Whole plant	Decoction	Wild	Dry or Fresh	Determined by the host plants	0.011	9	HYF171101006
67	Loranthaceae	*Taxillus chinensis* (DC.) Danser	Guang ji sheng 广寄生	Shrub	Whole plant	Decoction	Wild	Dry or Fresh	Tonifying liver and kidney, strengthening bones and muscles, lowering the blood pressure	0.032	202.5	HYF171219009
68	Loranthaceae	*Viscum multinerve* (Hayata) Hayata	Bing guo hu ji sheng 柄果槲寄生	Shrub	Whole plant	Decoction	Wild	Dry or Fresh	Tonifying liver and kidney, improving blood circulation, lowering the blood pressure	0.026	32.4	HYF180118011
69	Magnoliaceae	*Manglietia aromatica* (Dandy) V. S. Kumar	Xiang mu lian 香木莲	Tree	Fruit	Decoction	Wild	Dry	Regulating *qi* and invigorating consciousness	0.006	3.6	HYF180108032
70	Malvaceae	*Helicteres angustifolia* L	Shan zhi ma 山芝麻	Shrub	Whole plant	Decoction	Wild	Dry or Fresh	Clearing heat away and detoxifying	0.065	117	HYF180110009
71	Malvaceae	*Microcos paniculata* L	Po bu ye 破布叶	Tree	Leaf	Decoction	Wild	Dry or Fresh	Clearing heat away and detoxifying, anti-inflammatory, anti-diarrhea	0.529	1293.6	HYF180108037
72	Menispermaceae	*Cocculus laurifolius* DC	Zhang ye mu fang ji 樟叶木防己	Shrub	Leaf	Decoction	Wild	Dry or Fresh	–	0.013	8.1	HYF181120016
73	Menispermaceae	*Cyclea hypoglauca* (Schauer) Diels	Fen ye lun huan teng 粉叶轮环藤	Liana	Root	Decoction	Wild	Dry	Detoxifying, Anti-inflammatory	0.011	8.3	HYF181122004
74	Menispermaceae	*Pericampylus glaucus* (Lam.) Merr	Xi yuan teng 细圆藤	Liana	Leaf	Decoction	Wild	Dry	–	0.006	5.9	HYF190103019
75	Moraceae	*Ficus carica* L	Wu hua guo 无花果	Shrub	Fruit	Decoction	Cultivated	Dry	Invigorating stomach, aid digestion, detumescence, detoxification	0.393	1474.2	HYF180109020
76	Moraceae	*Ficus cyrtophylla* (Miq.) Miq	Wai ye rong 歪叶榕	Tree	Leaf	Decoction	Wild	Dry	–	0.011	5	HYF180109021
77	Moraceae	*Morus alba* L	Sang 桑	Tree	Leaf	Decoction	Cultivated	Dry	Clearing heat away, improving eyesight	0.592	2466	HYF180108043
78	Moraceae	*Morus australis* Poir	Ji sang 鸡桑	Shrub	Leaf	Decoction	Wild	Dry	Clearing heat away	0.119	61.9	HYF180108051
79	Myrtaceae	*Decaspermum gracilentum* (Hance) Merr. & L. M. Perry	Zi lian shu 子楝树	Tree	Leaf	Decoction	Wild	Dry	Diabetes, lowering the blood pressure, Hypolipidemic	0.022	7.8	HYF181122007
80	Myrtaceae	*Psidium guajava* L	Fan shi liu 番石榴	Tree	Young leaf	Decoction	Cultivated	Fresh	Anti-diarrhea, aid digestion	0.114	143.1	HYF190705022
81	Myrtaceae	*Syzygium nervosum* A. Cunn. ex DC	Shui weng pu tao 水翁蒲桃	Tree	Flower, young leaf	Decoction	Cultivated	Dry	Clearing heat away	0.054	39	HYF190507006
82	Nymphaeaceae	*Nelumbo nucifera* Gaertn	Lian 莲	Herb	Leaf	Decoction	Cultivated	Dry or Fresh	Clearing heat away, relieve summer heat	0.242	504	HYF171114009
83	Oleaceae	*Jasminum sambac* (L.) Aiton	Mo li hua 茉莉花	Liana	Flower	Soak	Cultivated	Dry or Fresh	–	0.654	1636.2	HYF160413005
84	Oleaceae	*Ligustrum robustum* (Roxb.) Blume	Cu zhuang nv zhen 粗壮女贞	Shrub	Leaf	Soak	Wild	Dry	Refreshing, dispelling wind and eliminating dampness, strengthening bones and muscles, lowering the blood pressure	0.017	5.3	HYF191112019
85	Oleaceae	*Ligustrum sinense* Lour	Duo mao xiao la 多毛小蜡	Shrub	Young leaf	Soak	Wild	Dry or Fresh	Relieving sore throat	0.009	0.7	HYF191112020
86	Oleaceae	*Osmanthus fragrans* Lour	Gui hua 桂花	Tree	Flower	Soak	Cultivated or wild	Dry or Fresh	Relieving cough and reducing sputum, improving eyesight	0.367	765	HYF190103017
87	Orchidaceae	*Anoectochilus calcareus* Aver	Hui yan jin xian lan 灰岩金线兰	Herb	Whole plant	Decoction	Wild	Dry	Clearing heat away, detoxification and detumescence, moistening lungs and relieving cough	0.114	178.9	HYF18111001
88	Orchidaceae	*Anoectochilus nandanensis* Y. Feng Huang & X. C. Qu	Nan dan jin xian lan 南丹金线兰	Herb	Whole plant	Decoction	Wild	Dry	Clearing heat away, detoxification and detumescence, moistening lungs and relieving cough	0.017	21.6	HYF18111002
89	Orchidaceae	*Anoectochilus roxburghii* (Wall.) Lindl	Hua ye kai chun lan 花叶开唇兰	Herb	Whole plant	Decoction	Wild	Dry	Clearing heat away, detoxification and detumescence, moistening lungs and relieving cough	0.32	1332	HYF171028002
90	Orchidaceae	*Anoectochilus zhejiangensis* Z. Wei & Y. B. Chang	Zhe jiang jin xian lan 浙江金线兰	Herb	Whole plant	Decoction	Wild	Dry	Clearing heat away, detoxification and detumescence, moistening lungs and relieving cough	0.002	1.6	HYF171028004
91	Orchidaceae	*Bulbophyllum kwangtungense* Schltr	Guang dong shi dou lan 广东石豆兰	Herb	Whole plant	Decoction	Wild	Fresh	Moistening lungs, relieving cough and reducing sputum, clearing heat away	0.099	165.6	HYF180211009
92	Orchidaceae	*Bulbophyllum odoratissimum* (Sm.) Lindl. ex Wall	Mi hua shi dou lan 密花石豆兰	Herb	Whole plant	Decoction	Wild	Fresh	Moistening lungs and resolving phlegm, relaxing tendons and activating collaterals	0.052	43.2	HYF180211010
93	Orchidaceae	*Nervilia fordii* (Hance) Schltr	Mao chun yu lan 毛唇芋兰	Herb	Whole plant	Decoction	Wild	Dry	Clearing heat away	0.039	52.7	HYF170413003
94	Orchidaceae	*Nervilia plicata* (Andrews) Schltr	Mao ye yu lan 毛叶芋兰	Herb	Whole plant	Decoction	Wild	Dry	Clearing heat away	0.009	3.1	HYF170413004
95	Orchidaceae	*Pholidota chinensis* Lindl	Shi xian tao 石仙桃	Herb	Whole plant	Decoction	Wild	Fresh	Moistening lungs, clearing heat away and detoxifying, eliminating dampness, dispersing stasis	0.616	2885.6	HYF190103022
96	Orchidaceae	*Pholidota pallida* Lindl	Yun nan shi xian tao 云南石仙桃	Herb	Whole plant	Decoction	Wild	Fresh	Clearing heat away, relieving cough and reducing sputum	0.14	438.8	HYF190103024
97	Pentaphylacaceae	*Adinandra millettii* (Hook. & Arn.) Benth. & Hook. f. ex Hance	Yang tong 杨桐	Shrub	Young leaf	Decoction	Wild	Dry	Anti-inflammatory, clearing heat away	0.13	81	HYF180201010
98	Pentaphylacaceae	*Adinandra nitida* Merr. ex H. L. Li	Liang ye yang tong 亮叶杨桐	Tree	Young leaf, flower	Soak	Wild	Dry	Detoxification, lowering the blood pressure, clearing heat away, health care	0.335	302.3	HYF180201012
99	Pentaphylacaceae	*Eurya chinensis* R. Br	Mi sui hua 米碎花	Shrub	Young leaf	Decoction	Wild	Dry or Fresh	Clearing heat away and detoxifying, preventing influenza	0.017	9.4	HYF180111002
100	Pentaphylacaceae	*Eurya patentipila* Chun	Chang mao ling 长毛柃	Shrub	Young leaf	Decoction	Wild	Dry or Fresh	–	0.013	2.3	HYF180109019
101	Phyllanthaceae	*Glochidion sphaerogynum* (Müll. Arg.) Kurz	Yuan guo suan pan zi 圆果算盘子	Shrub	Leaf	Decoction	Wild	Fresh	–	0.009	1.3	HYF180112013
102	Phyllanthaceae	*Phyllanthus emblica* L	Yu gan zi 余甘子	Tree	Fruit	Soak	Cultivated or wild	Fresh	Helping produce saliva and slake thirst, moistening lungs and resolving phlegm	0.212	496.1	HYF190103026
103	Pinaceae	*Pinus massoniana* Lamb	Ma wei song 马尾松	Tree	Leaf	Decoction	Wild	Dry	Improving blood circulation	0.194	273.4	HYF190103031
104	Plantaginaceae	*Plantago asiatica* L	Che qian 车前	Herb	Whole plant	Decoction	Wild	Fresh	Diuresis, relieving cough	0.646	4664.4	HYF190103033
105	Plantaginaceae	*Scoparia dulcis* L	Ye gan cao 野甘草	Herb	Whole plant	Decoction	Wild	Dry or Fresh	Decreasing blood glucose, lowering the blood pressure, antiviral and antitumor	0.259	1296	HYF171101005
106	Poaceae	*Bambusa chungii* McClure	Fen dan zhu 粉单竹	Tree	Young leaf	Decoction	Wild	Dry	–	0.112	79	HYF171028008
107	Poaceae	*Cymbopogon mekongensis* A. Camus	Qing xiang mao 青香茅	Herb	Leaf	Decoction	Cultivated	Dry	–	0.335	1255.5	HYF181122006
108	Poaceae	*Imperata cylindrica* (L.) Raeusch	Bai mao 白茅	Herb	Rhizome	Decoction	Wild	Dry or Fresh	Clearing away heat and diuresis	0.402	1674	HYF160413004
109	Poaceae	*Lophatherum gracile* Brongn	Dan zhu ye 淡竹叶	Herb	Whole plant	Decoction	Wild	Dry or Fresh	Clearing heat away, diuresis	0.104	216	HYF171117011
110	Poaceae	*Pogonatherum paniceum* (Lam.) Hack	Jin fa cao 金发草	Herb	Branch and leaf	Decoction	Wild	Dry	Clearing heat away and detoxifying	0.026	56.2	HYF190705017
111	Poaceae	*Saccharum officinarum* L	Gan zhe 甘蔗	Herb	Stem	Decoction	Cultivated	Fresh	Clearing heat away and detoxifying, help produce saliva and slake thirst, antiemetic	0.071	142.6	HYF151121017
112	Polygonaceae	*Polygonum chinense* (L.) H. Gross	Huo tan mu 火炭母	Herb	Branch and leaf	Decoction	Wild	Dry or Fresh	Clearing heat away, diuresis, detoxification, improving eyesight, improving blood circulation	0.035	56.3	HYF190705018
113	Primulaceae	*Maesa japonica* (Thunb.) Moritzi & Zoll	Du jing shan 杜茎山	Shrub	Young leaf	Decoction	Wild	Fresh	Clearing heat away and detoxifying	0.071	14.9	HYF180111054
114	Primulaceae	*Maesa montana* A. DC	Jin zhu liu 金珠柳	Shrub	Young leaf	Decoction	Wild	Fresh	–	0.091	56.7	HYF180111055
115	Primulaceae	*Maesa perlaria* (Lour.) Merr	Ji yu dan 鲫鱼胆	Shrub	Young leaf	Decoction	Wild	Fresh	–	0.108	22.5	HYF180111056
116	Pteridaceae	*Onychium japonicum* (Thunb.) Kunze	Ye zhi wei jin fen jue 野雉尾金粉蕨	Herb	Leaf	Decoction	Wild	Dry or Fresh	Detoxification, clearing heat away	0.147	106.1	HYF190103013
117	Rhamnaceae	*Berchemia polyphylla* Wall. ex M. A. Lawson	Dong ye gou er cha 多叶勾儿茶	Shrub	Branch and leaf	Decoction	Wild	Dry or Fresh	Clearing away the lung-heat	0.017	16.6	HYF171028008
118	Rhamnaceae	*Sageretia thea* (Osbeck) M. C. Johnst	Que mei teng 雀梅藤	Shrub	Branch and leaf	Decoction	Wild	Dry or Fresh	Clearing heat away and detoxifying	0.013	12.5	HYF190507003
119	Rosaceae	*Crataegus scabrifolia* (Franch.) Rehder	Yun nan shan zha 云南山楂	Tree	Leaf, fruit	Decoction	Wild	Dry	Aiding digestion	0.551	2151.6	HYF181120017
120	Rosaceae	*Docynia doumeri* (Bois) C. K. Schneid	Tai wan hai tang 台湾海棠	Tree	Fruit	Decoction	Wild	Dry	Aiding digestion	0.317	529.2	HYF180108031
121	Rosaceae	*Eriobotrya japonica* (Thunb.) Lindl	Pi pa 枇杷	Tree	Leaf	Decoction	Cultivated	Dry or Fresh	Relieving cough and reducing sputum	0.43	1343.3	HYF161104004
122	Rosaceae	*Rubus chingii* Hu	Tian cha 甜茶	Shrub	Leaf	Soak	Wild	Dry	Diuresis, lowering the blood pressure	0.199	496.8	HYF161114014
123	Rubiaceae	*Dimetia hedyotidea* (DC.) T. C. Hsu	Niu bai teng 牛白藤	Herb	Branch and leaf	Decoction	Wild	Dry or Fresh	Clearing heat away and detoxifying	0.026	42.2	HYF180110006
124	Rubiaceae	*Hedyotis caudatifolia* Merr. & F. P. Metcalf	Jian ye er cao 剑叶耳草	Herb	Branch and leaf	Decoction	Wild	Dry or Fresh	Relieving cough and reducing sputum, aid digestion	0.125	313.2	HYF180110002
125	Rubiaceae	*Hedyotis effusa* Hance	Ding hu er cao 鼎湖耳草	Herb	Branch and leaf	Decoction	Wild	Dry or Fresh	Clearing heat away and detoxifying	0.19	158.4	HYF180110004
126	Rubiaceae	*Hedyotis uncinella* Hook. & Arn	Chang jie er cao 长节耳草	Herb	Whole plant	Decoction	Wild	Dry or Fresh	Dispelling wind and eliminating dampness	0.013	18.7	HYF180110008
127	Rubiaceae	*Mycetia sinensis* (Hemsl.) Craib	Hua xian e mu 华腺萼木	Shrub	Branch and leaf	Decoction	Wild	Dry or Fresh	Help produce saliva and slake thirst	0.006	2.6	HYF171114008
128	Rubiaceae	*Uncaria hirsuta* Havil	Mao gou teng 毛钩藤	Liana	Flower	Soak	Wild	Fresh	Refreshing	0.009	3.9	HYF171219010
129	Rubiaceae	*Uncaria rhynchophylla* (Miq.) Miq	Gou teng 钩藤	Liana	Stem nodes with hooks	Decoction	Wild	Dry	Lowering the blood pressure, protection against the cold	0.013	9.4	HYF171219011
130	Rutaceae	*Citrus maxima* (Burm.) Merr	You 柚	Tree	Peel	Soak	Cultivated	Dry	Invigorating stomach, aid digestion, clearing away the lung-heat	0.086	96	HYF190312029
131	Rutaceae	*Micromelum minutum* (G. Forst.) Wight & Arn	Da guan 大管	Shrub	Leaf	Decoction	Wild	Dry or Fresh	Improving blood circulation	0.004	3.1	HYF180108039
132	Rutaceae	*Murraya tetramera* C. C. Huang	Si shu jiu li xiang 四数九里香	Shrub	Branch and leaf	Soak	Wild	Dry or Fresh	Dispelling wind and eliminating dampness	0.356	1782	HYF191112024
133	Santalaceae	*Viscum articulatum* Burm. f	Bian zhi hu ji sheng 扁枝槲寄生	Shrub	Whole plant	Decoction	Wild	Dry or Fresh	Clearing heat away and diuresis, dispelling wind and eliminating dampness	0.071	133.7	HYF180118009
134	Santalaceae	*Viscum liquidambaricola* Hayata	Feng xiang hu ji sheng 枫香槲寄生	Shrub	Whole plant	Decoction	Wild	Dry or Fresh	Clearing heat away and diuresis, dispelling wind and eliminating dampness	0.039	162	HYF180118010
135	Sapindaceae	*Dimocarpus longan* Lour	Long yan 龙眼	Tree	Aril	Soak	Cultivated	Dry	Nourish the brain, calm the nerves	0.328	684	HYF181122008
136	Saururaceae	*Houttuynia cordata* Thunb	Ji cai 蕺菜	Herb	Leaf	Decoction	Wild	Dry	Clearing heat away and detoxifying, diuresis	0.067	120.9	HYF180110014
137	Schisandraceae	*Illicium difengpi* B. N. Chang	Di feng pi 地枫皮	Shrub	Bark	Decoction	Wild	Dry	Dispelling wind and eliminating dampness	0.017	2.6	HYF191112023
138	Scrophulariaceae	*Buddleja officinalis* Maxim	Mi meng hua 密蒙花	Shrub	Flower	Soak	Wild	Dry	Clearing heat away, improving eyesight	0.162	168.8	HYF180211008
139	Theaceae	*Camellia euphlebia* Merr. ex Sealy	Xian mai jin hua cha 显脉金花茶	Shrub	Young leaf, flower	Soak	Wild	Dry or Fresh	Refreshing, clearing heat away and detoxifying	0.125	26.1	HYF1501106004
140	Theaceae	*Camellia flavida* Hung T.Chang	Dan huang jin hua cha 淡黄金花茶	Shrub	Flower	Soak	Wild	Dry or Fresh	Decreasing blood glucose, lowering the blood pressure, Hypolipidemic, lower cholesterol	0.119	12.4	HYF1501106005
141	Theaceae	*Camellia huana* T. L. Ming & W. J. Zhang	Gui zhou jin hua cha 贵州金花茶	Shrub	Flower	Soak	Wild	Dry or Fresh	Decreasing blood glucose, lowering the blood pressure, Hypolipidemic, lower cholesterol	0.065	15.2	HYF1501106006
142	Theaceae	*Camellia impressinervis* H. T. Chang & S. Ye Liang	Ao mai jin hua cha 凹脉金花茶	Shrub	Flower	Soak	Wild	Dry or Fresh	Decreasing blood glucose, lowering the blood pressure, Hypolipidemic, lower cholesterol	0.024	5.6	HYF1501106007
143	Theaceae	*Camellia indochinensis* Merr	Dong xing jin hua cha 东兴金花茶	Shrub	Flower	Soak	Wild	Dry or Fresh	Decreasing blood glucose, lowering the blood pressure, hypolipidemic, lower cholesterol	0.017	4.1	HYF1501106008
144	Theaceae	*Camellia petelotii* (Merr.) Sealy	Jin hua cha 金花茶	Shrub	Flower, young leaf	Soak	Cultivated or wild	Dry or Fresh	Decreasing blood glucose, lowering the blood pressure, hypolipidemic, lower cholesterol	0.194	81	HYF1501106009
145	Theaceae	*Camellia pubipetala* Y. Wan & S. Z. Huang	Mao ban jin hua cha 毛瓣金花茶	Shrub	Flower	Soak	Wild	Dry or Fresh	Decreasing blood glucose, lowering the blood pressure, hypolipidemic, lower cholesterol	0.032	7.6	HYF1501107002
146	Theaceae	*Stewartia sinensis* Rehder & E. H. Wilson	Zi jing 紫茎	Tree	Young leaf	Decoction	Wild	Dry or Fresh	Improving blood circulation	0.011	1.7	HYF190507004
147	Theaceae	*Stewartia villosa* Merr	Rou mao zi jing 柔毛紫茎	Shrub	Young leaf	Decoction	Wild	Dry or Fresh	Improving blood circulation	0.006	1	HYF190507005
148	Urticaceae	*Pilea sinofasciata* C. J. Chen	Cu chi leng shui hua 粗齿冷水花	Herb	Whole plant	Decoction	Wild	Dry or Fresh	Clearing heat away and detoxifying, regulating qi	0.175	273.4	HYF190103029
149	Vitaceae	*Nekemias cantoniensis* (Hook. & Arn.) J. Wen & Z. L. Nie	Guang dong she pu tao 广东蛇葡萄	Liana	Branch and leaf	Decoction	Wild	Dry	Clearing heat away, relieve summer heat	0.017	16.6	HYF160114005
150	Vitaceae	*Nekemias grossedentata* (Hand.-Mazz.) J. Wen & Z. L. Nie	Xian chi she pu tao 显齿蛇葡萄	Liana	Branch and leaf	Soak	Wild	Dry	Clearing heat away and detoxifying, dispelling wind and eliminating dampness	0.361	1202.4	HYF171012003
151	Vitaceae	*Vitis flexuosa* Thunb	Ge shu pu tao 葛藟葡萄	Liana	Branch and leaf	Decoction	Wild	Dry	–	0.019	7	HYF151121012
152	Vitaceae	*Vitis lanceolatifoliosa* C. L. Li	Ji zu pu tao 鸡足葡萄	Liana	Branch and leaf	Decoction	Wild	Dry	–	0.017	7.2	HYF151121014
153	Zingiberaceae	*Alpinia officinarum* Hance	Gao liang jiang 高良姜	Herb	Leaf	Decoction	Wild	Dry	Protection against the cold	0.013	19.4	HYF160114003
154	Zingiberaceae	*Alpinia zerumbet* (Pers.) B. L. Burtt & R. M. Sm	Yan shan jiang 艳山姜	Herb	Leaf	Decoction	Wild	Dry or Fresh	Expelling wind and removing cold	0.004	5.4	HYF160114008
155	Zingiberaceae	*Zingiber officinale* Roscoe	Jiang 姜	Herb	Tuber	Decoction	Cultivated	Fresh	Clearing heat away, antiemetic, relieving cough	0.745	3105	HYF151121015

#### Family distribution

**Fig. 3:**
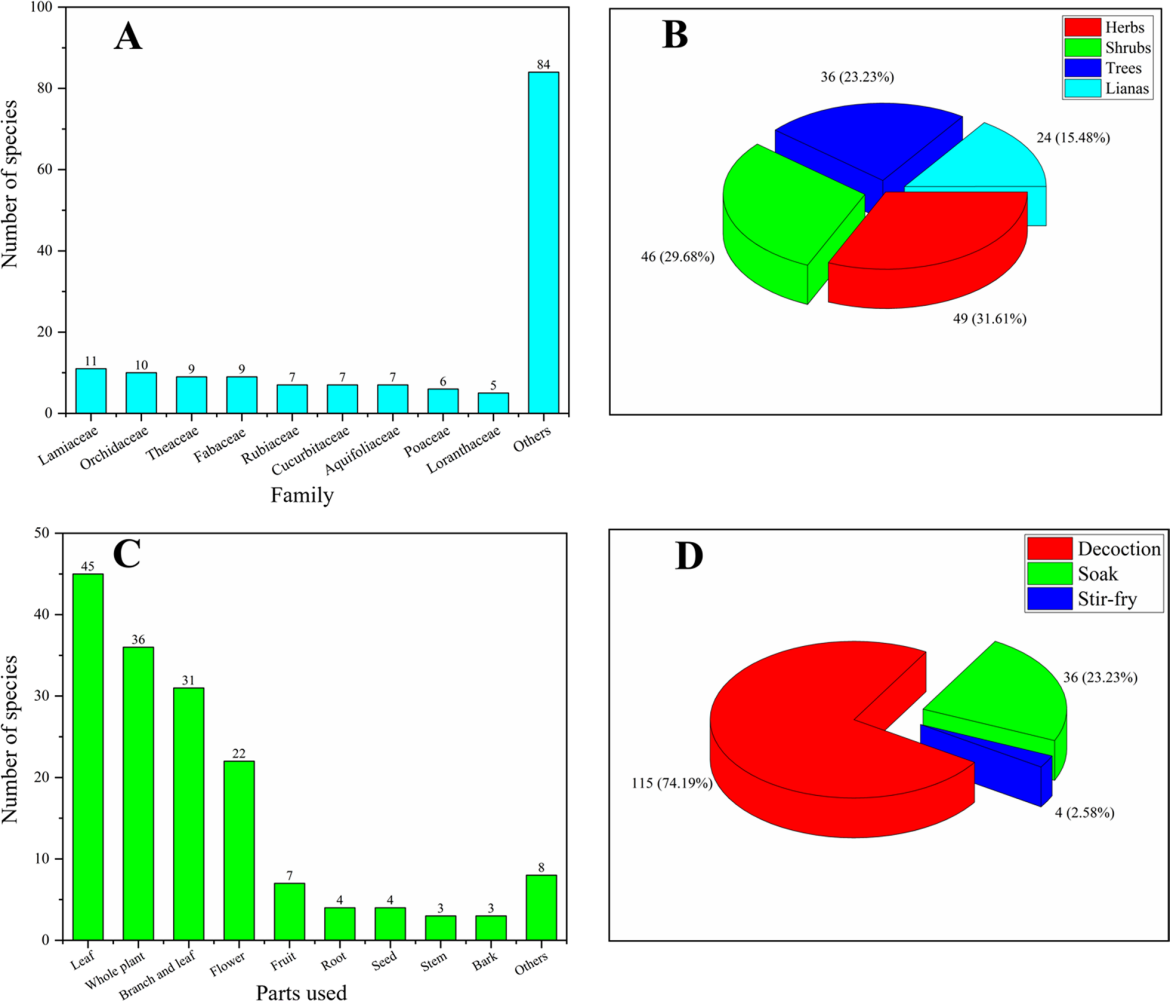
**A** Family distribution of herbal tea species; **B** Life form of herbal tea species; **C** Use parts of herbal tea species; **D** Preparation methods of herbal tea species

The most frequently used families were Lamiaceae (11 species), Orchidaceae (10 species), Theaceae (9 species), Fabaceae (9 species), Rubiaceae (7 species), Cucurbitaceae (7 species), Aquifoliaceae (7 species), Poaceae (6 species), Loranthaceae (5 species), and other 40 families contributing 84 species are represented mainly by four or fewer entities (Fig. 3A).

#### Habit and habitat of herbal tea

For the habit of 155 herbal tea species, the most frequent species were herbs, represented by 49 species, followed by shrubs with 46 species, trees with 36 species, and lianas with 24 species (Fig. 3B). In addition, most of them (124 species, 80%) were obtained from wild habitats, whereas only 20 (12.9%) species were cultivated, and 11 (7.09%) species were wild or cultivated. Similar findings were reported by other studies from China [11, 23]. Local people believe that wild plants are healthier than cultivated ones. In addition, they prefer dry materials because they believe that it would taste better than fresh ones. Also, dry materials are easier to store and more readily available when guests visiting.

#### Parts used

Local people in Guangxi use different plant parts to prepare herbal tea, and two parts can be used in some species for tea preparing (Table 1). The leaf was the most commonly used part, represented by 45 species, followed by whole plant with 36 species, branch and leaf 31 species, flower 22 species, and fruit 7 species (Fig. 3C). Other plant parts, including seed, root, bark, tuber, peel, and rhizome, are used less frequently. Leaves are more accessible in people’s daily lives. They are more likely to be tested by humans for the first time and learn from other animals’ behavior. Some herbal tea varieties were made from young leaves because they are similar in shape to *Camellia sinensis*, such as *Adinandra nitida*, *Eurya chinensis*, and *Maesa japonica*. This is one of the reasons for leaves was the most commonly used plant part of herbal tea [11, 22].

#### Preparation methods and materials status of herbal tea

Different plant parts may subject to different preparation methods for herbal tea drinks make. Three different modes of preparation were documented in this study. Decoction was the most commonly used processing method, represented by 115 species, followed by soak with 36 species. Four species (*Chamaecrista mimosoides*, *Chamaecrista nictitans*, *Senna sophera*, and *Senna tora*) were used soak after stir-fry (Fig. 3D). Some parts like stems, whole plants, barks, and old leaves are often processed by decoction, but young leaves and flowers are preferable to soak. The decoction is widely used in rural areas, while urban populations prefer the soak. Some herbal tea, especially cooling tea, can be served with sugar by urban people. Conversely, rural inhabitants prefer to drink the herbal tea without adding anything else. Most herbal tea preparations involved using single plant species or a single plant part, such as the stems of *Neocinnamomum delavayi* was cooked as herbal tea to prevent cold and cure infantile diarrhea, treat most distinguished guest, and ceremony festival by Zhuang people in Napo County, western Guangxi, while other parts of this species were not used as herbal tea in this area. According to our investigation and documentation, only a few herbal tea varieties were used to mix with traditional tea (*Camellia sinensis*), such as *Jasminum sambac*, *Zingiber officinale*, and *Osmanthus fragrans*, to obtain special aroma and taste. In the UK, Ireland, Canada, and India, milk is typically added into tea, while it is more common to take tea with lemon and honey in Eastern Europe. Several studies have shown that preparation conditions greatly affect the amount of extracted bioactive compounds such as polyphenols [38, 39].

#### Health-promoting effects and ICF of herbal tea

Various health-promoting effects of herbal tea consumption have historically been recognized by Chinese people [40]. Based on our investigation, a total of 141 herbal tea species have auxiliary efficacy, which is over ninety percent of our reported herbal tea in this study. Clearing heat away was the most common auxiliary efficacy, followed by detoxifying, improving blood circulation, cold and cough, tonic, and aid digestion (Table 2). Moreover, other auxiliary efficacies were expressed in a few numbers of herbal tea, such as alleviating a hangover, anti-inflammatory, antiviral, antitumor, calming the nerves, refreshing, anti-diabetes, treating headache, helping saliva producing and slake thirst, regulating *qi*, relaxing tendons, and activating collaterals (Table 2).

**Table 2:** Informant consensus factor by categories of health-promoting effects in the study area

Category	Specific conditions (number of species)	Nur	Nt	ICF
Clearing heat away	Clearing heat away (82), relieving summer heat (11), clearing away the lung-heat (2), clearing liver heat (3), heatstroke prevention (1), expelling wind and removing cold (2)	101	45	0.56
Detoxifying	Detoxifying (44), detumescence (7)	51	30	0.42
Improving blood circulation	Cholesterol-lowering (7), hypolipidemic (7), decreasing blood glucose (8), lowering the blood pressure (18), dispersing stasis (1)	39	17	0.58
Tonic	Health care (1), improving eyesight (9), improving immunity (1), invigorating stomach (2), moistening lungs (12), nourishing the brain (1), protecting against the cold (3), tonifying liver and kidney (2), strengthening bones and muscles (2)	34	10	0.73
Removing cold and cough	Removing cold (3), relieving cough (18), resolving phlegm (2), reducing sputum (7), relieving sore throat (1), expectorant (1)	32	11	0.68
Eliminating dampness and diuresis	Dispelling wind and eliminating dampness (8)	8	3	0.71
Aiding digestion	Anti-diarrhea (4), antiemetic (2)	6	1	1.00
Others	Alleviate a hangover (1), anti-inflammatory (3), antiviral and antitumor (1), calm the nerves (1), refreshing (4), diabetes (1), headache (1), help produce saliva and slake thirst (5), regulating qi (1), relaxing tendons and activating collaterals (1)	19	6	0.72

Forty-one diseases reported by the informant were divided into eight categories. The ICF values for all disease types ranged from 0.42 to 1 (Table 2). The kind of disease with highest in ICF was the aiding digestion (1.00), followed by the tonic (0.73), eliminating dampness and diuresis (0.71), removing cold and cough (0.68), improving blood circulation (0.58), and clearing heat away (0.56). The high value of ICF for aid digestion, tonic, and eliminating dampness and diuresis may be due to the limited number of reports and information. The Nur and Nt of tonic (34, 10), removing cold and cough (32, 11), improving blood circulation (39, 17), and clearing heat away (101, 45) were all relatively high, indicating that local people had high consistency in these health-promoting effects of herbal tea.

### Evaluation of herbal tea based on RFC and CFSI values

**Fig. 4:**
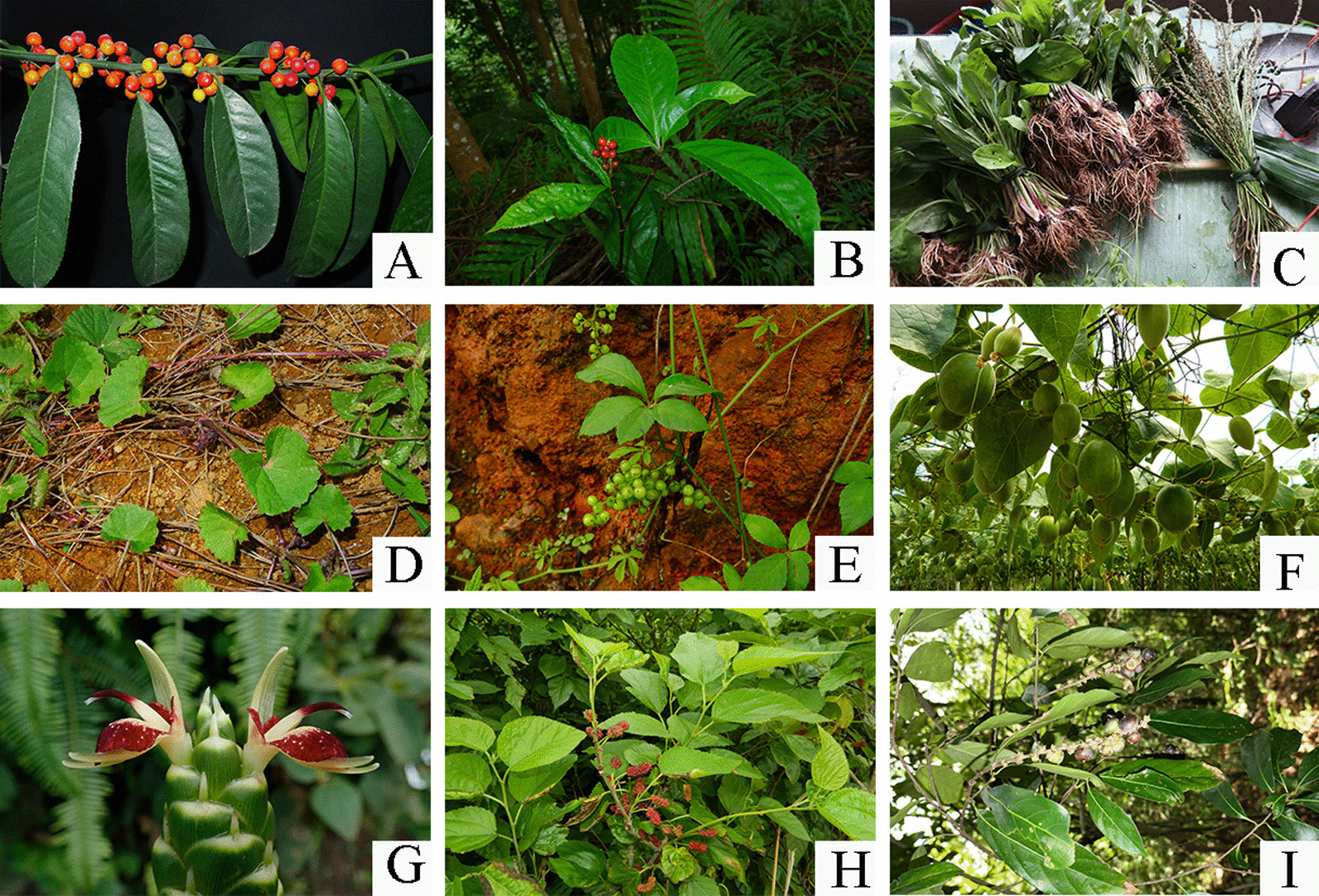
Some herbal tea plants. **A** *Ilex kudingcha*; **B** *Sarcandra glabra*; **C** *Plantago asiatica*; **D** *Centella asiatica*; **E** *Gynostemma pentaphyllum*; **F** *Siraitia grosvenorii*; **G** *Zingiber officinale*; **H** *Morus alba*; **I** *Lithocarpus litseifolius*

Relative frequency of citation (RFC) and cultural food significance index (CFSI) were applied to evaluate the important herbal tea in this study (Table 3). RFC reflects the relative importance of certain plants in a particular area. The RFC values of all herbal tea ranged from 0.002 to 0.773, among which the highest one was *Siraitia grosvenorii* (0.773), followed by *Zingiber officinale* (0.745) and *Chrysanthemum indicum* (0.704) (Table 1). The values of the cultural food significance index (CFSI) varied considerably from one species to another, with a minimum of 0.7 and a maximum of 5370.0. The most culturally significant herbal tea species were *Siraitia grosvenorii* (5370.0), *Plantago asiatica* (4664.4), *Gynostemma pentaphyllum* (3244.8), *Zingiber officinale* (3105.0), *Pholidota chinensis* (2885.6), and *Morus alba* (2466.0) (Table 3). Some species used for herbal tea are displayed in Fig. 4, and the details in the calculation of CFSI for each species are provided in Additional file 2: Table S2.

**Table 3:** Evaluation of herbal tea plants using CFSI (> 1000) and RFC index

Species	Indices		Ranking	
	CFSI	RFC	CFSI	RFC
*Siraitia grosvenorii* (Swingle) C. Jeffrey ex A. M. Lu & Zhi Y. Zhang	5370.0	0.773	1	1
*Plantago asiatica* L	4664.4	0.646	2	5
*Gynostemma pentaphyllum* (Thunb.) Makino	3244.8	0.562	3	9
*Zingiber officinale* Roscoe	3105.0	0.745	4	2
*Pholidota chinensis* Lindl	2885.6	0.616	5	7
*Morus alba* L	2466.0	0.592	6	8
*Crataegus scabrifolia* (Franch.) Rehder	2151.6	0.551	7	10
*Centella asiatica* (L.) Urb	1958.4	0.294	8	34
*Murraya tetramera* C. C. Huang	1782.0	0.356	9	23
*Sarcandra glabra* (Thunb.) Nakai	1689.6	0.380	10	17
*Imperata cylindrica* (L.) Raeusch	1674.0	0.402	11	15
*Jasminum sambac* (L.) Aiton	1636.2	0.654	12	4
*Ficus carica* L	1474.2	0.393	13	16
*Artemisia anomala* S. Moore	1408.3	0.05	14	89
*Ilex kudingcha* C. J. Tseng	1380.6	0.637	15	6
*Lithocarpus litseifolius* (Hance) Chun	1377.0	0.551	16	10
*Eriobotrya japonica* (Thunb.) Lindl	1343.3	0.430	17	14
*Anoectochilus roxburghii* (Wall.) Lindl	1332.0	0.320	18	31
*Scoparia dulcis* L	1296.0	0.259	19	36
*Microcos paniculate* L	1293.6	0.529	20	12
*Cymbopogon mekongens* A. Camus	1255.5	0.335	21	26
*Ampelopsis grossedentata* (Hand.-Mazz.) J. Wen & Z. L. Nie	1202.4	0.361	22	21
*Platostoma palustre* (Blume) A. J. Paton	1117.8	0.149	23	47
*Orthosiphon aristatus* (Blume) Miq	1017.4	0.339	24	25

#### Special and representative herbal tea in Guangxi

In addition to the herbal tea plants selected by the index, there are some particular and representative herbal tea plants in Guangxi, such as *Adinandra nitida*, *Neocinnamomum delavayi*, and *Hedyotis effusa*.

The young leaves of *A. nitida* are commonly used as *Shiya* tea (石崖茶) among rural communities. However, according to our investigation, some Yao people also collect its flower buds to make herbal tea, with clearing and detoxifying effects, and restraining and sterilizing bacteria. Prices vary enormously from buds to leaves. The flower buds are much more expensive. Currently, the complexity of abstraction and refined productions of buds are rare. The best time for collecting *A. nitida* is from middle May to early June. The brief preprocessing is as follows: firstly, dry the buds of *A. nitida* in the sun for one day or so, then bring them out of the direct sunlight for 2 or 3 weeks at a cool, well-ventilated place. It is light yellow color and intense flower fragrance, and a full-flavored palate that is unique yet smooth, with a memorable aftertaste.

The leaves of *Neocinnamomum delavayi* are common ingredients of Chinese herbal remedies to treat wind–dampness arthralgia syndrome, bruises, and wounds bleeding effectively. For Zhuang people lived in Pingmeng Town, Napo County, western Guangxi, the local people cut the stems into several pieces, then put them in a pan and cook, occasionally stirring, until red and just cooked for 4 to 5 min. This tea is used for a ceremony by the Zhuang people. The gift of hospitality is dedicated to the most distinguished guests. According to the villagers, the tea can prevent from getting cold and cure infantile diarrhea. However, current phytochemical research on *N. delavayi* is mainly focused on the chemical components of volatiles extracted from leaves. The pharmacological activity of this plant and its role in the human body are ignored.

*Hedyotis effusa*, also known as a Longgougan, is a medicinal plant in Fangchenggang and Qinzhou, which is easy to find in the variety of medicinal markets. The population of *H. effusa* once puzzled and fascinated us for a long time. Therefore, an efforted interview with the local people was conducted. According to the interviews, inhabitants are predisposed to get inflamed by the damp and muggy climate, boiling *H. effusa* for a tasty way to beat every summer’s heat.

#### Comparison of herbal tea between Guangxi and other neighboring areas

**Fig. 5:**
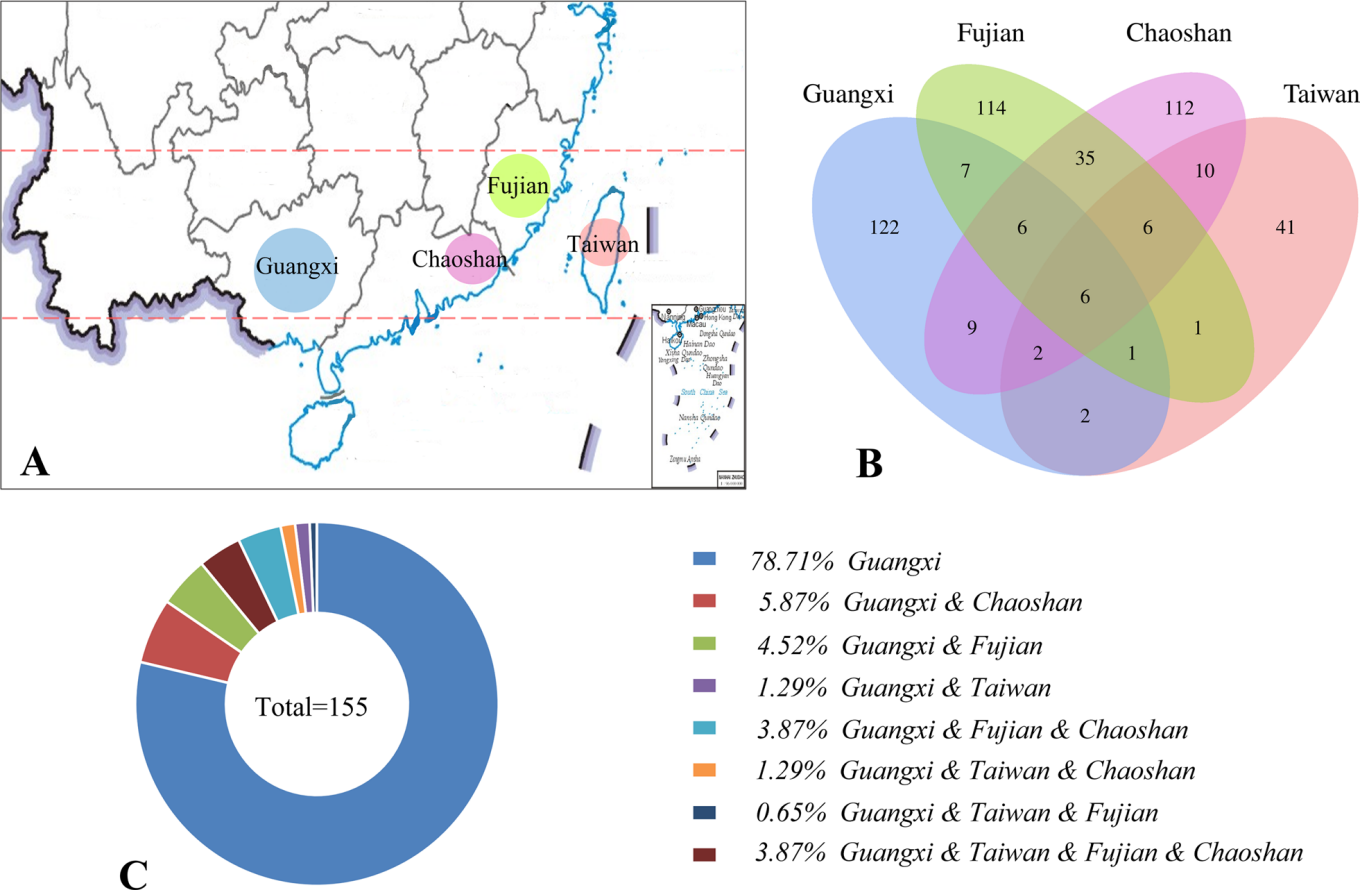
Comparison of herbal tea species between Guangxi and other areas in China. **A** The geographic distribution of the compared regions; **B** Venn diagram for the comparison of the plant species from different regions; **C** proportions of overlapping plant species used between Guangxi and neighbored regions

Herbal tea or cooling tea drinks were popular in Southern China and widely used for healthcare due to the damp humidity and heat levels of this area. In addition, rich cultural diversity of Southern China was presented with numerous Chinese minorities distributed in this zone. Therefore, to illustrate whether the geographical and cultural difference affected the choice and use of herbal tea species in Guangxi, we compared the species in our study with previous investigated herbal tea materials in Chaoshan [26], Fujian [27], and Taiwan [28] (Fig. 5A).

A Venn diagram was made to visualize herbal tea species consumed in four places. The results showed that there were 9 species both in Guangxi and Chaoshan, 7 species in Guangxi and Fujian, and 2 species in Guangxi and Taiwan (Fig. 5B). Moreover, there are 6 plant species (*Centella asiatica*, *Houttuynia cordata*, *Imperata cylindrica*, *Morus alba*, *Plantago asiatica*, and *Prunella vulgaris*) used among the four-place comparison (Fig. 5B). It is proposed that these species grow in these compared regions due to the similar natural environment conditions, and benefit to the local people’s health on preventing or treating common diseases in similar environment and climate. Remarkably, 122 (78.71%) of the 155 raw materials were used only in Guangxi (Fig. 5B–C), indicating that Guangxi also has its own special selection of herbal tea raw materials based on the unique composition of ethnic minorities and culture.

## Discussion

### Healthcare effects and safety of herbal tea consumption in Guangxi

Various health-promoting effects of herbal tea consumption have been historically recognized by Chinese people [40]. In this study, the most frequently mentioned healthcare functions of herbal tea were to clear heat away, represented by 101 species (65.16%). Similar results were found in other studies [3, 9, 21, 22]. “Heat” is an important medical term in Traditional Chinese Medicine (TCM) and various ethnomedical systems in China [41]. It is a pathogenic syndrome in the human body and may lead to a range of human health problems such as influenza, fever, cough, dizziness, and lung abscess [42, 43]. To “clearing heat away and detoxifying” is critical and frequently terms in TCM, which is equally to prevent or treat heat-related symptoms, and to treat infections from viruses and bacteria or the poisoning caused by food, heavy metals, and pesticide. *Ilex kudingcha*, *Gynostemma pentaphyllum*, *Hypericum japonicum*, and *Microcos paniculata* were widely used as a Liáng chá (“cooling tea” or “cool tisane” in Chinese) in Southern China [21, 44–47]. Herbal tea consumption has been considered an important element of traditional medicine that focuses on preventive therapies and treating sub-health conditions through targeted dietary changes, mood management, and a work rest balance [48, 49]. Herbal tea-drinking habit plays an important role in traditional healthcare system in Guangxi. Forty-one herbal teas could improve blood circulation, thirty-three could be used for tonic, and six could aid digestion. Some studies have reported that herbal tea has great potential in preventing and treating chronic metabolic diseases [50–56].

Herbal tea is often consumed safely by people without any restriction on the dosage that has a long history [11]. Although few adverse events associating with the most frequently mentioned herbal teas were found in our investigation, caution should be taken as “natural” is not always good. Fu et al. reported that some herbal teas' overconsumption might cause negative effects [11]. Other studies also found that some phytochemicals in herbal tea are risky to humans [11, 57–61]. The content and quality of herbal tea products must be controlled under the related legal requirement throughout the supply chain from collection, transportation, processing, production, and storage. New technologies and methods, such as two-dimensional chromatography fingerprinting, molecular identification, and chemical detection, should be developed to detect chemical contaminants and adulterants of herbal tea plant species [62–64]. Policies and administrative management about herbal tea products and the formulation of their quality standard may ensure their manufacture following the legal requirements. Public awareness of potential safety issues associated with herbal tea products must also be improved through propaganda and education programs.

### Local cultural differences could affect the choice of herbal tea plants

Herbal tea or cooling tea drinks are popular in Southern China and widely used for healthcare due to the damp humidity and heat levels of this area. Previous ethnobotanical studies have documented the plant materials and related traditional knowledge of herbal tea used in a few areas located in Southern China, such as Chaoshan [26], Fujian [27], and Taiwan [28]. In this study, a comparison of herbal tea between Guangxi and three neighboring areas (Chaoshan, Fujian, and Taiwan) was made. The results indicated that Guangxi has its own unique selection of herbal tea species. However, these compared four places have similar latitude ranges in geographical location (Fig. 5A) and hot/humid subtropical monsoon climate, which should result in similar natural environment conditions among these places. It means the natural environment is not the reason or at least the main reason for the unique choice of herbal tea plants by local people in Guangxi. Given this, the population composition and corresponding specific culture could be proposed as a crucial reason for the choice of herbal tea species.

As an autonomous region, Guangxi has the largest minority population in China. The Zhuang nationality accounts for 83.28% of minority population and 31.36% of the population in Guangxi [25]. In addition, the ethnic groups, including Yao (3.7%), Miao (1.1%), Dong (0.7%), Mulam (0.4%), and Maonan (0.17%), have sizable populations in Guangxi [25]. The Hakka, belonging to Han branch speaking Hakka dialects, has settled down in Chaoshan, Fujian, and Taiwan with a very considerable population [27, 29, 30]. Therefore, based on the above population composition of the compared places, the traditional knowledge of the main ethnic groups such as Zhuang, Yao, and Miao, and their culture on the use of plant resource could be one of the reasons for the differences in herbal tea species used compared to the other three areas, whose selection of herbal tea species may be affect by the traditional knowledge and culture from local communities. Importantly, it is necessary to further investigate how do the local culture affects the choice of herbal tea plants in the future.

### Herbal tea is facing increasing opportunities and challenges

In urban areas of Guangxi, small stores run liáng chá was very popular here and there. The liáng chá industry has dramatically grown around Guangxi to meet regional, national, and global demand for herbal tea and dietary supplements for part reason of Guangxi government promotion [65]. This phenomenon is in line with the modern pursuit of health and dietary requirements. This active demand will certainly result in increased herbal tea. On the one hand, the sustainability of the herbal drinks' ethnomedicinal base is threatened with global environmental change, expanded commercialization, policies, and over-harvesting of natural resources. On the other hand, it promotes the cultivation of herbal tea plants to develop better and faster. For example, *Camellia petelotii*, as an herbal tea, has been listed as one of the most endangered species in China due to its natural population size [66]. Recent pharmacological studies revealed that this plant has good healthcare functions for its rich bioactive components [67]. In the past, it was not used extensively because of restrictions on wild natural resources, and the price was too high (the highest point reaching 30 000 CNY per kilogram) [68]. Advanced technology-based breeding and cultivation made *C. petelotii* becoming common in recent decades. Similarly, *Gynostemma pentaphyllum*, *Ilex kudingcha*, and *Adinandra nitida* have also begun industrialization for orientating markets on brand extensions.

## Conclusions

This study conducted a comprehensive ethnobotanical investigation across Guangxi to document the plant species used as herbal tea, traditional knowledge of the herbal tea including used parts, preparation and treatments, and analysis of the cultural significance, health consistency, and special characteristics of Guangxi herbal tea. Our study recorded 155 herbal tea species in Guangxi. Most of these species were herbaceous plants, most commonly used part was leaf, and the main preparation method was decoction. Moreover, forty-one health benefits were reported from the recorded herbal tea and clearing heat away was the most common health-promoting effect. In total, 122 herbal tea species were only found in Guangxi compared to the herbal tea species reported in neighbored regions; among them, *Siraitia grosvenorii*, *Plantago asiatica*, *Gynostemma pentaphyllum*, *Zingiber officinale*, *Pholidota chinensis*, and *Morus alba* were the most cultural significance herbal tea species in Guangxi.

Our findings revealed that local people have rich traditional knowledge about herbal tea, which plays a vital role in their healthcare. These traditional knowledge and culture could affect the local people to select and use different herbal tea plants. The recorded herbal tea species in this study possess tremendous potential for local economic development in the future. Further research on efficacy evaluation and product development of herbal tea species is necessary.

## Supplementary Information

**Additional file 1**. The information of surveyed villages and markets.

**Additional file 2**. The detailed values of CFSI for each species.

## Data Availability

The data, materials, and information are acquired from the manuscript and supplementary materials. The others out of manuscript and supplementary will be made available upon request to authors.
